# The characteristics of collagen‐induced rheumatoid arthritis in macaques and the changes of heart

**DOI:** 10.1002/ame2.70185

**Published:** 2026-03-17

**Authors:** Lei Zhang, Haifeng Jiang, Zhen Xu, Tingyu Dong, Xiaoyi Liu, Shangxue Yan, Jiajie Kuai, Yan Chang, Wei Wei

**Affiliations:** ^1^ School of Pharmacy and Science Anhui Medical University Hefei Anhui China; ^2^ Key Laboratory of Anti‐inflammatory and Immune Medicine, Ministry of Education Anhui Medical University Hefei Anhui China; ^3^ Institute of Clinical Pharmacology Anhui Medical University Hefei Anhui China; ^4^ Department of Pharmacy The First Affiliated Hospital of Anhui Medical University, Anhui Public Health Clinical Center Hefei Anhui China

**Keywords:** collagen‐induced arthritis, non‐human primates, rheumatoid arthritis

## Abstract

**Background:**

Based on human Rheumatoid Arthritis (RA) criteria, this study established a macaque collagen‐induced arthritis (CIA) model and a standardized non‐human primate (NHP) standardized diagnostic framework. Integrating these tools, we characterized three disease stages (IIR, e‐RA, a‐RA) and key early biomarkers, thereby providing a robust platform for investigating RA pathogenesis research and conducting preclinical evaluation of humanized macromolecular drugs.

**Methods:**

CIA was induced in macaques via through immunization with bovine type II collagen emulsified in complete Freund's adjuvant. Disease progression was monitored using multimodal imaging (MRI, CT, X‐ray, and ultrasonography), enabling comprehensive assessment of joint edema, vascularity, bone erosion, and cardiopulmonary function.

**Results:**

The disease progression in CIA macaques was categorized into three stages: IIR (days 14–34), e‐RA (days 35–41), and a‐RA (days 42–70). Notably, bone erosion progressed independently of the resolution of joint swelling resolution, with a trend toward aggravated severity during the post‐swelling phase. Furthermore, cardiac assessments demonstrated that valvular damage primarily affected the tricuspid valve, with more pronounced impairment of right ventricular diastolic function. Additionally, observed pulmonary inflammation suggests potential lung involvement, though its progression throughout the disease course requires further investigation.

**Conclusions:**

The progression of CIA in macaques can be divided into three stages: IIR (14–34 days), e‐RA (35–41 days), and a‐RA (42–70 days). For RA patients, bone erosion should be prioritized in clinical monitoring, and cardiac assessment should focus on the tricuspid valve and right ventricular diastolic function.

## Graphical abstract

The disease progression in CIA macaques was categorized into three stages: *IIR (days 14–34)*: The indicators related to rheumatoid arthritis and the levels of immunoglobulin were all elevated. There was a small amount of joint effusion, edema and mild synovial hyperplasia. No joint swelling was observed by naked eye. The overall score was 3–5 points. The aortic valve of the heart showed enhanced echo. *e‐RA (days 35–41)*: The ACPA level has significantly increased, joint effusion has become more prominent, and synovial hyperplasia has worsened. Joint swelling can be observed with the naked eye, and the overall score is 6–7 points. The aortic valve of the heart shows an increase in echo intensity, tricuspid valve regurgitation occurs, and diastolic function changes. *a‐RA (days 42–70)*: Bone erosion occurs, with peak levels of RF, ACPA and immunoglobulin. Joint swelling worsens and is accompanied by systemic symptoms (redness and swelling of the nose, nodules on the tail). The overall score is 7–9 points. The tricuspid valve regurgitation significantly increases. Notably, bone erosion progressed independently of joint swelling resolution, with a trend toward aggravated severity in the post‐swelling phase.

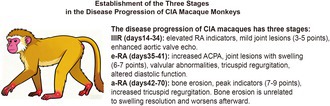

## 1 INTRODUCTION

Rheumatoid arthritis (RA) is a chronic autoimmune disease that is characterized by inflammatory in changes of the synovial tissue of joints, cartilage, and bone. In European and American countries, the prevalence of RA is about 0.2%–0.78%, and in China, it is about 0.28%.^1^ At present, most RA researchers focus on rodent models. However, due to inherent morphological and physiological differences between humans and rodents, rodent models do not faithfully recapitulate the pathogenesis of humans, and drug efficacy observed in these models is often difficult to verify clinically. An article published in the journal *Nature* in 2014 called drugs evaluated in rodents “rat drugs”.^2^ Non‐human primates (NHP) have the closest genetic background, phenotypic characteristics, and immunological characteristics to humans, so they can be more effectively used to understand the occurrence and development of human diseases and to develop research on prevention and treatment measures. They offer a translationally superior model for biomedical research, enabling faithful recapitulation of immune pathogenesis. This unique advantage effectively bridges the translational gap inherent in rodent models of immune‐mediated diseases.^3^, ^4^

Current research on NHP models of RA has primarily focused on fundamental model development, identification of specific serological markers, and preliminary therapeutic efficacy assessments. However, given the complex pathogenesis of RA and its progression through distinct clinical stages, there is a critical and urgent need to establish well‐characterized NHP models that enable systematic evaluation of disease staging and temporal patterns of onset and progression. This is particularly important as treatment initiation in most RA patients is frequently delayed until the active stage, leading to prolonged suffering, worsened prognoses, and irreversible joint damage. Therefore, NHP models that recapitulate early‐stage RA changes are essential for facilitating early detection and timely intervention. The 2010 diagnostic criteria jointly established by the European League Against Rheumatism (EULAR) and the American College of Rheumatology (ACR) represent a significant advance, incorporating four domains—affected joints, serological markers, acute‐phase reactants, and symptom duration—with a score of ≥6 confirming RA diagnosis. These criteria offer improved sensitivity for early RA detection, while the 1987 ACR criteria remain in use for diagnosing active‐stage disease. Further refining this paradigm, EULAR introduced the concept of Pre‐Rheumatoid Arthritis (Pre‐RA) in 2016, outlining a multi‐stage continuum of RA development. This model begins with genetic and environmental risk factors, progresses to a systemic autoimmunity stage—characterized by detectable autoantibodies in asymptomatic individuals, also referred to as the inflammatory immune response (IIR) stage—and may transition through undifferentiated arthritis (early RA, e‐RA), and culminates in definitive RA (active RA, a‐RA).^5^

Based on established human diagnostic and staging criteria for RA, this study developed a standardized laboratory assessment framework for NHP models of RA. We seek to characterize the progression through the IIR, e‐RA, and a‐RA stages by defining their temporal and pathological features, monitoring early biomarkers, and implementing integrated cardiopulmonary imaging. The findings are anticipated to yield crucial insights into RA disease mechanisms and accelerate the advancement of humanized biologic therapies.

## 2 METHODS

### 2.1 Animals

Twelve adult male macaques (weighing 5–8 kg, aged 3–4 years old) were used in this study. All animals were purchased from the South Anhui Macaque Domestication and Breeding Cooperative in Jingde County, Anhui Province (license number: SCXK (Anhui) 2020‐001). Throughout the study, the animals were maintained under controlled environmental conditions: an environmental temperature of 21°C–26°C, 40%–70% humidity, and a 12:12 h light/dark cycle. The animals were housed individually in stainless steel cages and randomly assigned into 2 groups: 4 animals were used as controls, and 8 animals were used as the model group. The macaques were acclimated to laboratory conditions for at least 4 weeks. They were fed with fresh vegetables and fruits once a day, and commercial chow twice a day (Beijing Keao Xieli Feed Co., Ltd). Water was provided ad libitum. The animals were anesthetized via intramuscular injection of Zoletil50 (a mixture of Tiletamine hydrochloride and zolazepam hydrochloride, VIRBAC, France) at a dose of 4–6 mg/kg before examination. The ulcerative skin lesions at the immunization site were cleaned with saline and iodine, and mupirocin ointment (Sino‐US Tianjin Shike Pharmaceutical Co., Ltd) was applied for infection control. The names of the macaques are listed in part 1.1 of the Supplementary Materials.

The research adhered to the principles of China's Laboratory Animal Care Guidelines and was approved by the Ethics Review Committee for Animal Experimentation of the Institute of Clinical Pharmacology, Anhui Medical University (approval number: PT‐2020‐001).

### 2.2 Induction of CIA

Bovine type II collagen (CII) (4 mg/mL, Chondrex, Washington, USA) was used. The CII solution and Freund's complete adjuvant (FCA) (Chondrex, Washington, USA) were mixed in equal proportions using a double‐syringe method. Each macaque was anesthetized by intramuscular injection of 10 mg/kg ketamine and intradermally injected with 2 mL of the emulsion at the back and tail root. The second immunization with CII and FCA was conducted 21 days after the first immunization, following the same protocol. The scoring criteria can be found in Table S1.

### 2.3 Ultrasound, computed tomography (CT), and magnetic resonance imaging (MRI) examination

Ultrasound (Vetus, Mindray, China) was used on D0, D28, D35, D42, and D49 to observe synovial hyperplasia, blood flow, heart valve regurgitation, and echocardiographic parameters (including E/A ratios) in all macaques. Joint edema and bone erosion were observed by Magnetic Resonance Imaging (MRI, MAGNZTOM ESSENZA 1.5T, SIEMENS, Germany) and Computed Tomography (CT, TSX‐101A, TOSHIBA, Japan) on days D0, D28, D35, D42, D49, D56, and D100 (i.e., 7 weeks after the second immunization). X‐rays were taken on D0, D42, D49, D56, D63, D70, and D100.

### 2.4 Other methods

Other methods are described in the Supplementary Materials.

## 3 RESULTS

### 3.1 Establishment of the three stages (IIR, e‐RA, a‐RA) in the disease progression of CIA macaques

Based on the early diagnostic criteria for RA proposed by EULAR and ACR in 2010 (Table S1), we established diagnostic criteria for RA in laboratory animals, including the following indicators: joint swelling, rheumatoid factor (RF), anti‐citrullinated protein antibodies (ACPA), erythrocyte sedimentation rate (ESR), C‐reactive protein (CRP), and systemic scores. Joint edema, bone erosion, and blood flow signals were observed using MRI, CT, and ultrasound. A total score of ≥6 was defined as a positive RA diagnosis (Table S2).

#### 3.1.1 Establishment of the e‐RA stage in CIA macaques

**FIGURE 1:**
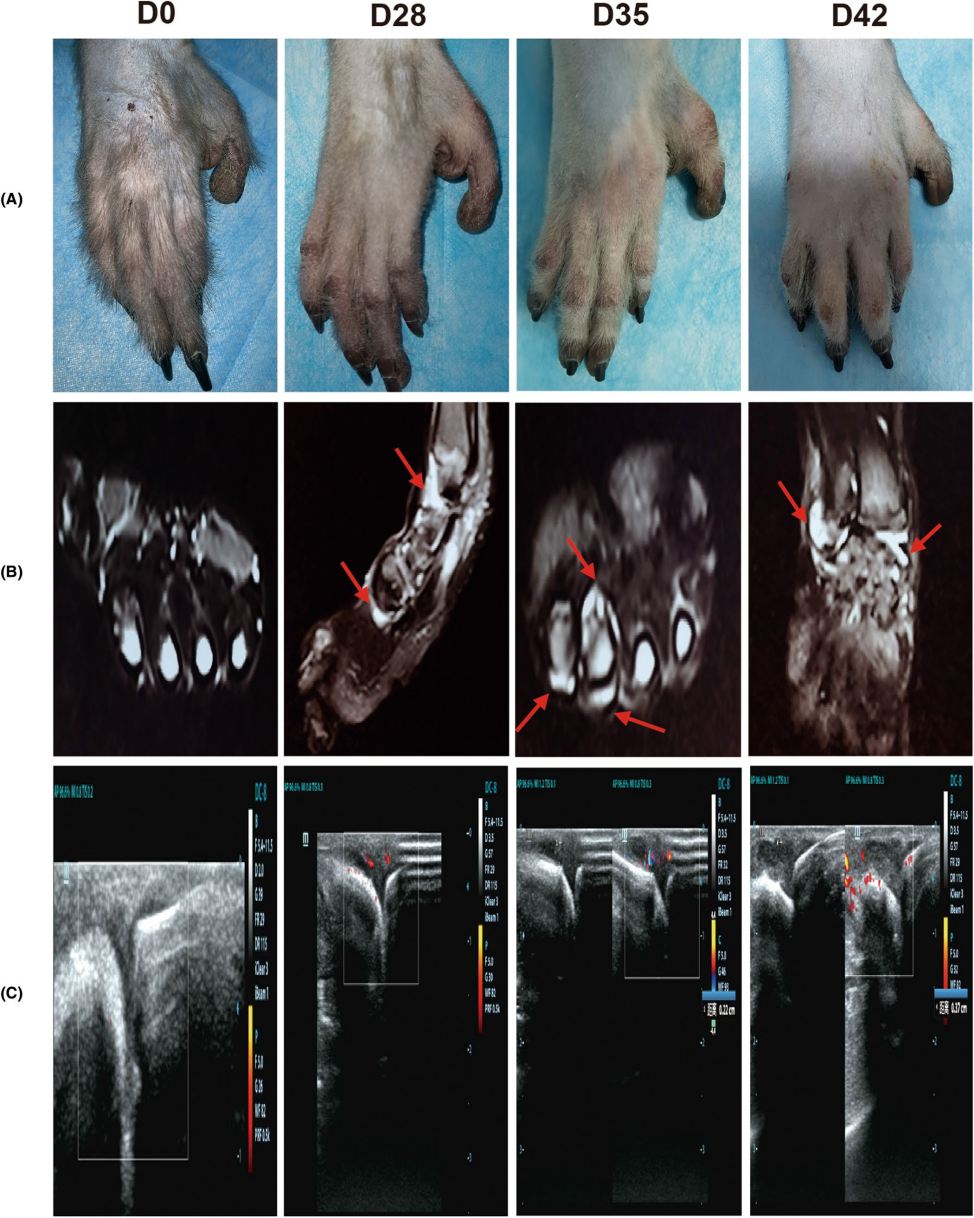
Joint swelling, edema, synovial hyperplasia, and blood flow signal changes in CIA macaques on D0, D28, D35, and D42. (A) Toe swelling degree. (B) Fluid accumulation observed by MRI; red arrows indicate joint effusion. (C) Synovial hyperplasia and blood flow signals observed by ultrasound.

**FIGURE 2:**
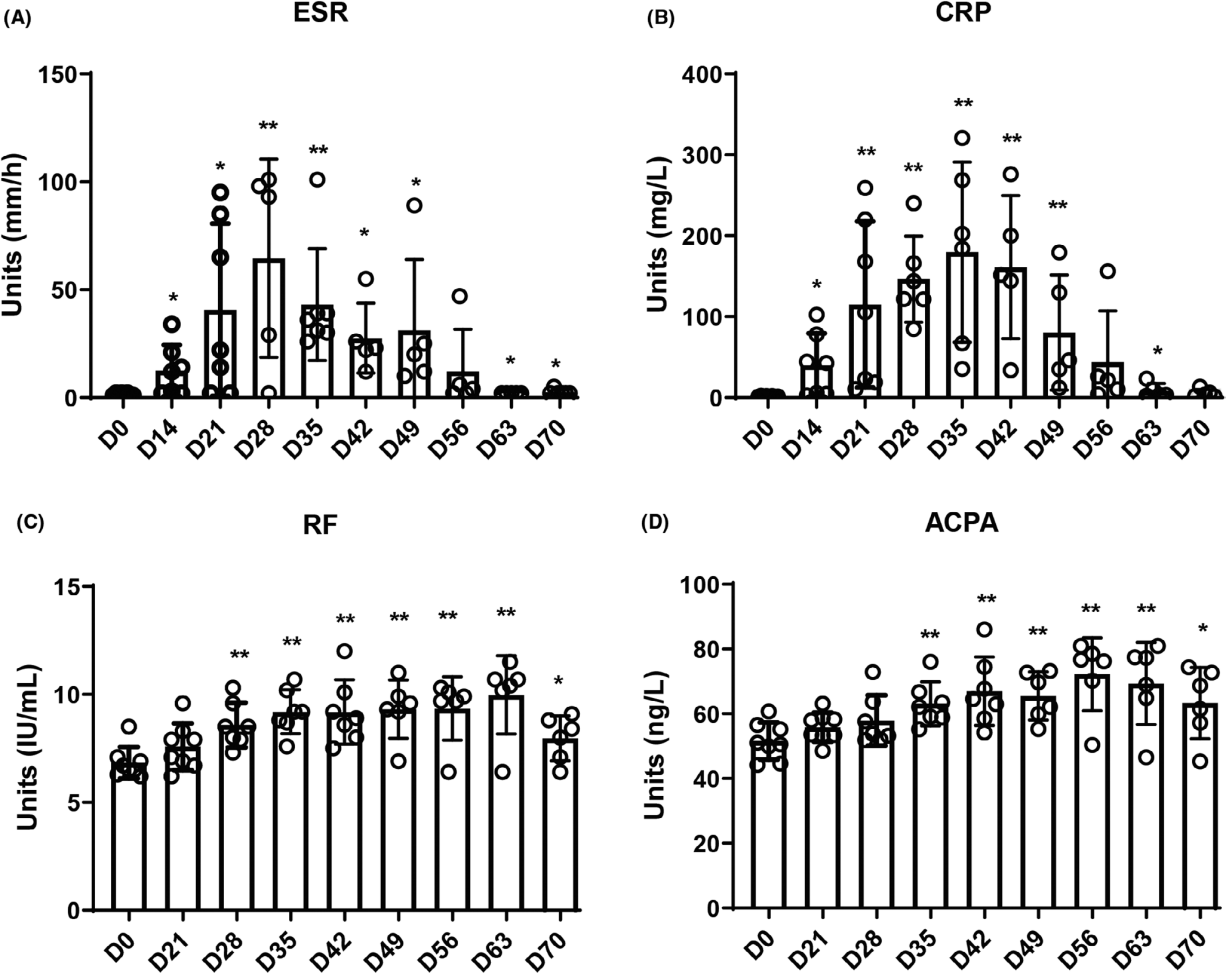
Bar chart showing the changes in peripheral blood rheumatoid‐related diagnostic indicators and memory response substances levels in CIA macaque at different times. (A) Erythrocyte sedimentation rate. (B) C‐reactive protein. (C) Rheumatoid factor. (D) Anti‐citrullinated protein antibodies. *p < 0.05, **p < 0.01 compared with the D0 group.

According to the diagnostic criteria for RA in NHP animals, we evaluated CIA macaques based on four aspects: affected joints, serology, acute‐phase reactants, and systemic scores. The results showed that no obvious toe swelling was observed on day 28 (Figure 1A). MRI and ultrasound results on D28 showed joint effusion, edema, and mild synovial hyperplasia (Figure 1B,C), and ESR, CRP, and RF levels were significantly elevated (Figure 2A–C). The total scores of the CIA group did not exceed 6 (Table 1), suggesting that this stage was still part of an inflammatory immune developmental period.

**TABLE 1:** Overall scoring results of CIA macaques.

Date	Name							
	TT	ZZ	YY	XX	LL	TF	GZ	RF
D0	0	0	0	0	0	0	0	0
D14	1	1	1	1	0	1	1	1
D21	1	3	3	1	3	3	3	3
D28	4	3	5	3	3	3	3	—
D35	6	6	7	6	6	6	4	—
D42	7	7	8	7	7	7	5	—
D49	9	8	—	8	8	7	5	—
D56	9	8	—	8	8	7	5	—
D63	8	8	—	8	7	7	3	—
D70	6	6	—	6	5	5	3	—

*Note*: “—” indicates sacrifice; RF and YY were sacrificed on D23 and D44, respectively.

Further observation showed that joint swelling increased significantly on D35, with aggravated joint effusion, edema, and synovial hyperplasia (Figure 1A–C), and ACPA levels rose markedly (Figure 2D). Except for the CIA macaque that was sacrificed on D23 (due to abnormal RF levels), 6 out of the remaining 7 macaques scored ≥6 (Table 1). Based on these results, we determined that D35 marked the onset of the e‐RA stage.

#### 3.1.2 Establishment of the IIR stage in CIA macaques

**FIGURE 3:**
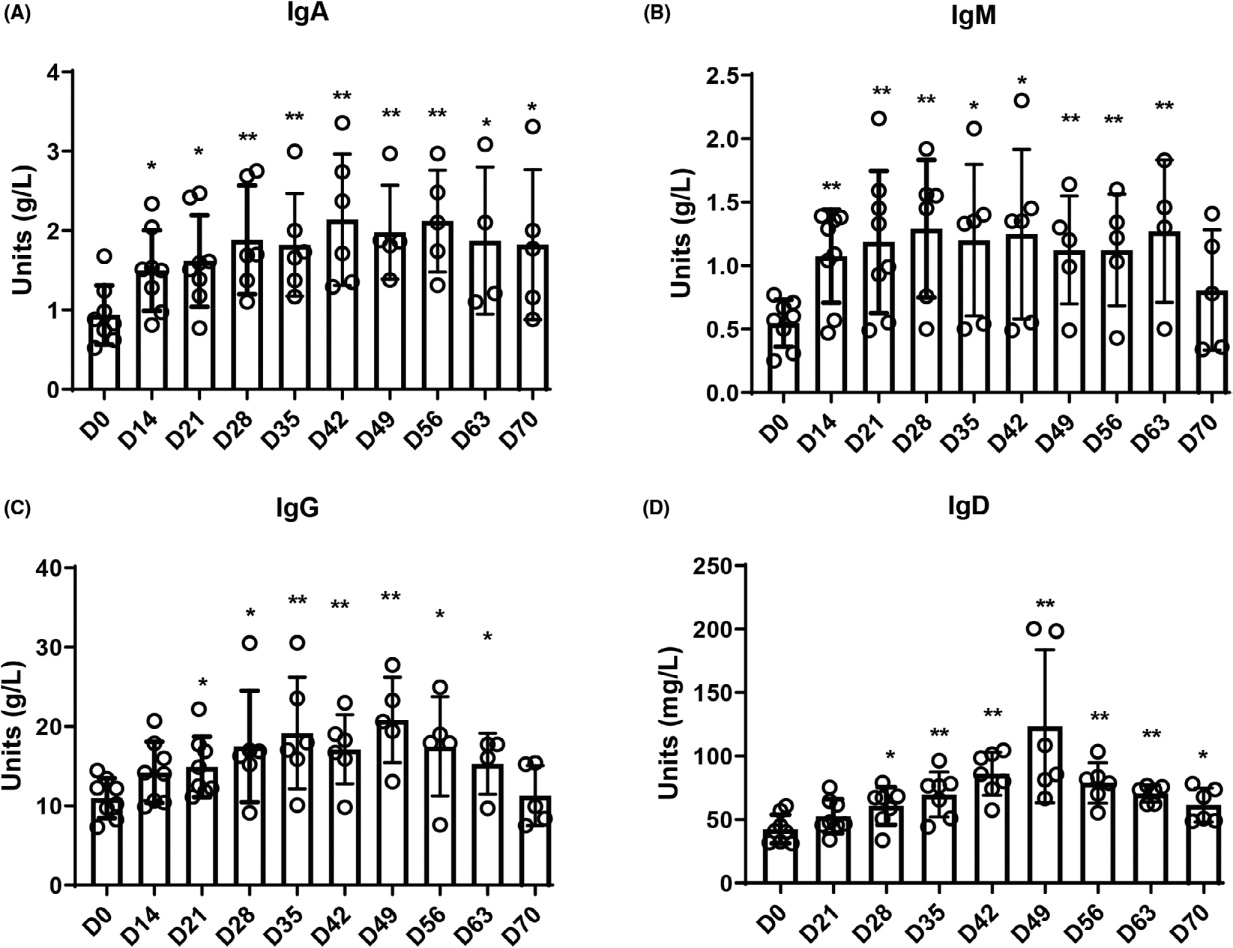
Changes in peripheral blood serum immunoglobulin levels of CIA macaques at different times. (A) Immunoglobulin A. (B) Immunoglobulin M. (C) Immunoglobulin G. (D) Immunoglobulin D. *p < 0.05, **p < 0.01 compared with the D0 group.

After determining D35 as the onset of e‐RA, the period before D35 was defined as the IIR stage. To identify the start of the IIR stage, we observed that ESR and CRP levels began to rise significantly from D14, peaking on D28 and D35, respectively (Figure 2A,B). Immunoglobulin A (IgA) and Immunoglobulin M (IgM) levels increased significantly from D14 (Figure 3A,B), while Immunoglobulin G (IgG) and Immunoglobulin D (IgD) levels rose from D21 and D28, respectively (Figure 3C,D). These inflammatory and immune response indicators continued to change until after D35, which we identified as the onset of e‐RA. Therefore, we concluded that D14–D34 represented the IIR stage in CIA macaques. Due to experimental limitations, inflammatory and immunological indicators on D7 were not measured. This study focused on early immunological changes during RA progression, particularly after the second immunization (D21).

#### 3.1.3 Establishment of the a‐RA stage in CIA macaques

**FIGURE 4:**
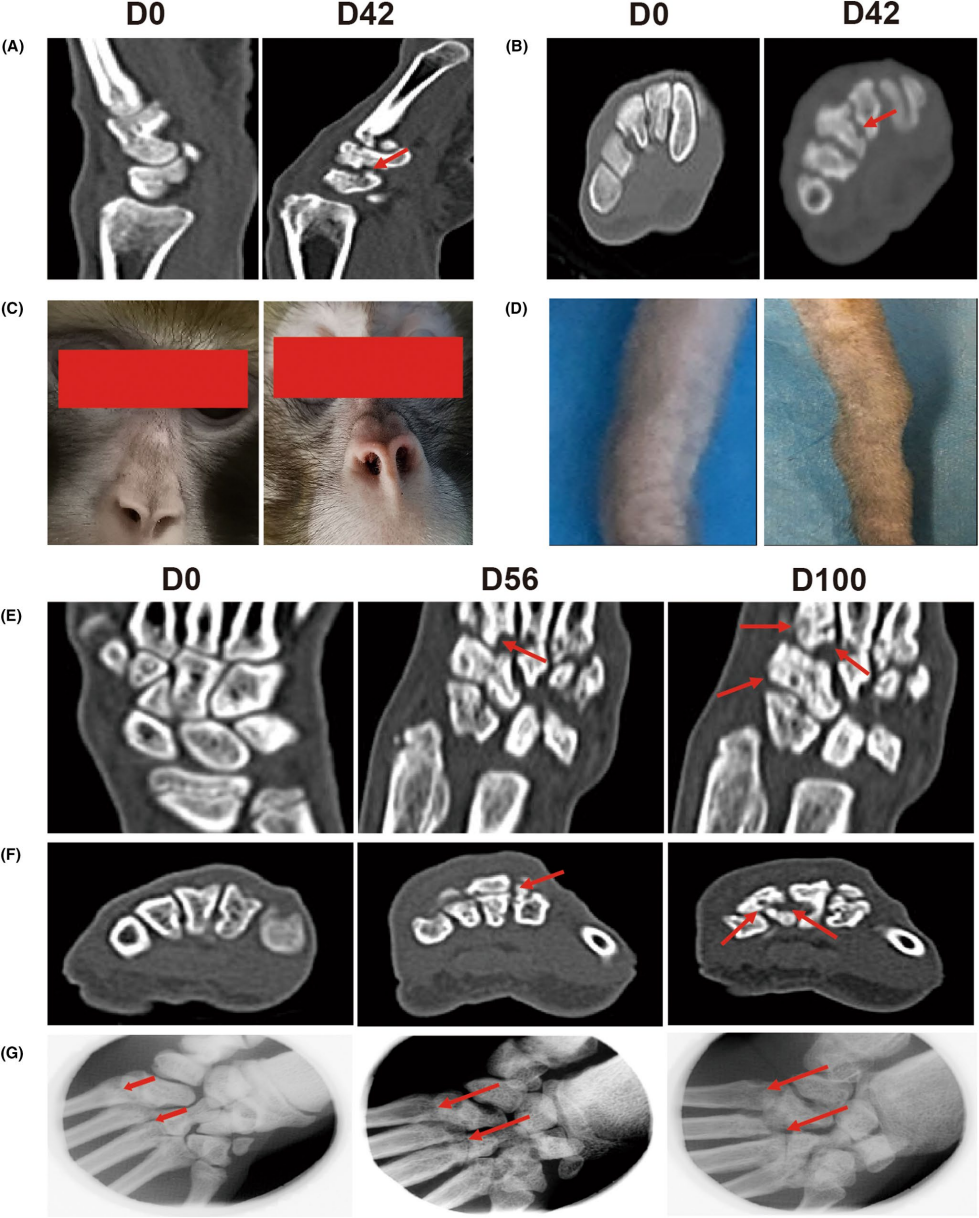
Nasal and tail swelling and bone erosion in CIA macaques at different time points. (A) Sagittal bone erosion in the wrist on D0 and D42 observed by CT. (B) Transverse bone erosion in the toe joints on D0 and D42 observed by CT. (C) Nasal redness and swelling on D0 and D42. (D) Tail swelling on D0 and D49. (E) Coronal bone erosion in the wrist on D0, D56, and D100 observed by CT. The red arrow indicates bone erosion. (F) Transverse bone erosion in the wrist on D0, D56, and D100 observed by CT. The red arrow indicates bone erosion. (G) Joint space observations on D0, D56, and D100 by X‐ray. The red arrow indicates the femoral gap.

According to the ACR 1987 diagnostic criteria for RA, the most notable change between e‐RA and a‐RA is the appearance of bone erosion. CT observations revealed bone erosion in the wrist and toe joints of CIA macaques starting from D42 (Figure 4A,B). Gross observations revealed that macaques exhibited red and swollen noses on D42, while tail nodules were observed on D49 (Figure 4C,D). Based on these results, we determined that the a‐RA stage began on D42. Further observation revealed that CRP and ESR returned to nearly normal levels by D70 (Figure 1A,B). RF and ACPA levels started to decrease significantly from D63 and D56, respectively, and approached normal levels by D70 (Figure 1C,D). Among the four immunoglobulins, only IgA and IgD remained elevated, while the other two returned to near‐normal levels. Individual macaque scores peaked on D56, and by D70 only three macaques scored ≥6 (Table 1). Based on these findings, we concluded that the a‐RA stage in CIA macaques spanned from D42 to D70.

#### 3.1.4 Characteristics of bone erosion in CIA macaques

**FIGURE 5:**
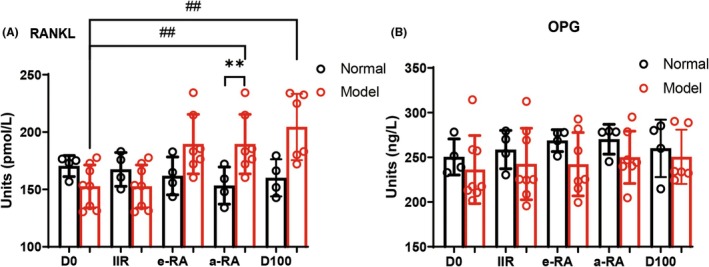
The changes in the levels of peripheral blood bone destruction‐related cytokines in macaques at different stages. (A) Receptor activator of nuclear factor kappa‐B ligand. (B) osteoprotegerin. ^##^ *p* < 0.01 compared with the D0 Model group; **p < 0.01 compared with the a‐RA Normal group.

CT observations revealed bone erosion in CIA macaques starting from D42. A comparison of bone erosion in the wrist joints on D56 and D100 showed no improvement; in fact, the condition worsened (Figure 4E,F). X‐ray observations also indicated rough joint surfaces and progressive narrowing of joint spaces (Figure 4G). In addition, we measured the levels of the bone destruction‐related factors receptor activator of nuclear factor kappa‐B ligand (RANKL) and osteoprotegerin (OPG) in the peripheral blood. The level of RANKL began to rise at the e‐RA stage, reached its peak at the a‐RA stage, and remained at a relatively high level until D100 (Figure 5A). The level of OPG did not change significantly (Figure 5B). This suggests that although joint swelling subsided after the active RA phase, bone erosion persisted and even worsened, indicating its irreversible nature.

### 3.2 Changes in cardiac function and characteristics of myocardial and vascular injury in macaques

**FIGURE 6:**
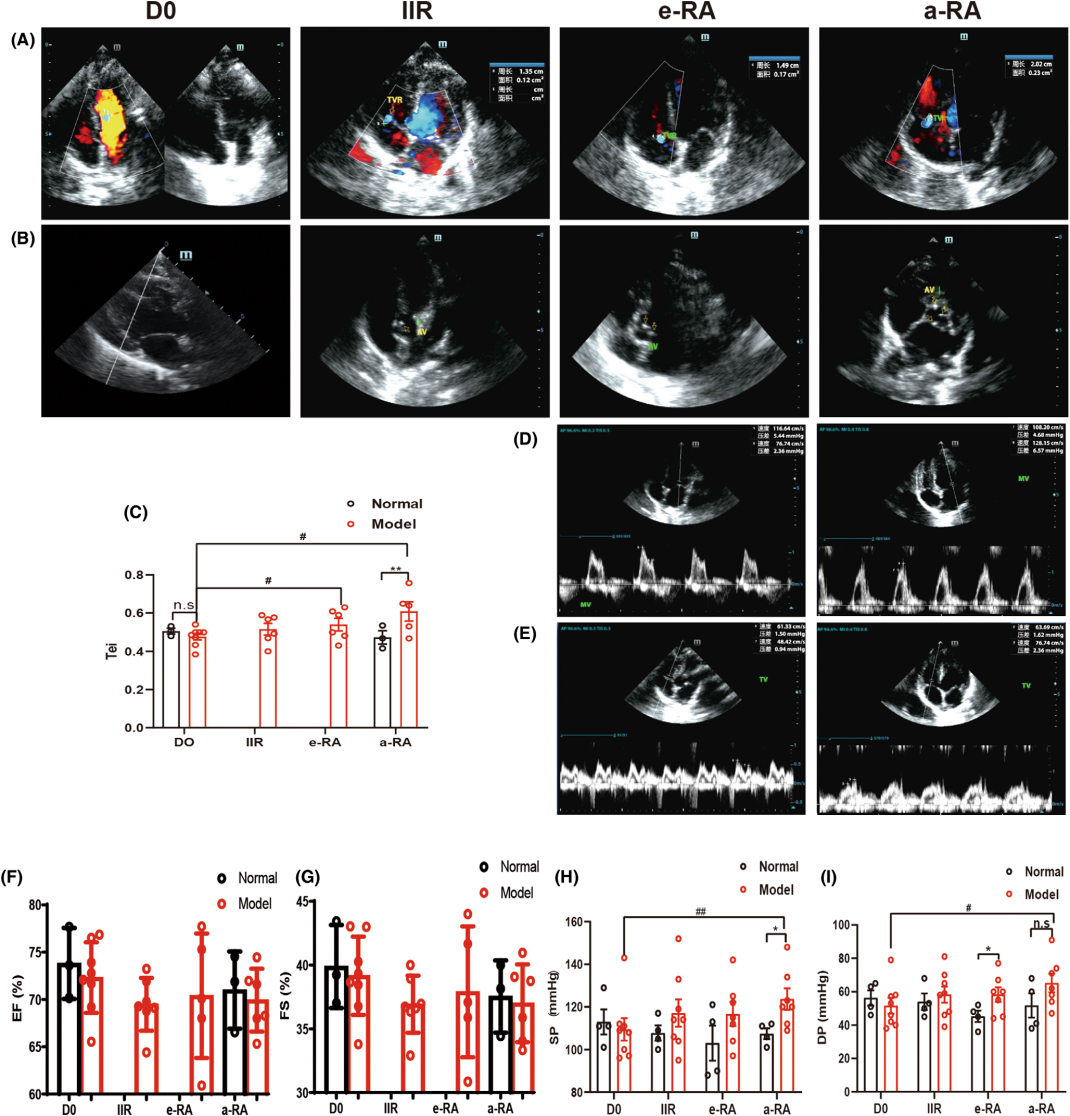
The changes of blood pressure, heart function and pulmonary inflammation observed by automatic ECG, ultrasound and CT in CIA macaques. (A) Tricuspid regurgitation results in CIA macaques at different stages. (B) The echo enhancement results of the aortic valve in CIA macaques at different stages. In color Doppler echocardiography, red indicates blood flow toward the probe and blue indicates flow away from the probe. In the apical four‐chamber view, red signals represent forward atrioventricular flow through the mitral and tricuspid valves. The blue jet at the tricuspid valve orifice suggests tricuspid regurgitation (TVR). Scattered blue regions away from the valve orifice likely reflect mild turbulence due to high heart rate. Blue signals within the left ventricle in the IIR and a‐RA stages indicate normal systolic outflow toward the aorta. (C) Results of blood pressure and cardiac Tei in CIA macaques at different stages (Tei = (IVCT + IVRT)/AET)). (D) Normal (left) and abnormal (right) representation of mitral valves in model group. (E) Normal (left) and abnormal (right) representation of tricuspid valves in model group. (F) Ejection fraction. (G) Fractional shortening. (H) Systolic blood pressure. (I) Diastolic blood pressure. ^#^ *p* < 0.05, ^##^ *p* < 0.01 as compared with the D0 Model group. *p < 0.05, **p < 0.01 as compared with the a‐RA Normal group.

Ultrasound was used to observe cardiac diastolic and systolic function, valvular regurgitation, and echo enhancement. The echocardiography examination confirmed the presence of valve damage, which was manifested by an increase in the echo of at least one group of valve leaflets, accompanied by varying degrees of blood reflux at the valve orifice.^6^, ^7^ The area of mitral regurgitation in all macaques on D0 was ≤0.14 cm^2^, the area of tricuspid regurgitation was ≤0.16 cm^2^, and there was no physiological regurgitation of the aortic valve. Thus, we defined the following as positive signals: mitral regurgitation area >0.14 cm^2^, tricuspid regurgitation area >0.16 cm^2^, and no aortic regurgitation as positive signals. In CIA macaques, three (TT, ZZ, LL) had two or more valve regurgitations (Table S3). Tricuspid valve involvement was the most frequently observed characteristic (Figure 6A, Table S3). Both normal and CIA macaques mitral valves showed enhanced echogenicity. We defined the echogenic signals of newly emerging tricuspid and aortic valves as positive signals. In CIA macaques, 4 (TT, ZZ, XX, YY) showed enhanced echo from 2 or more valves (Table S4). Echocardiographic evaluation demonstrated progressive enhancement of valvular echoes with disease progression, with predominant involvement of the aortic and tricuspid valves (Figure 6B, Table S4). The overall functional indicator Tei index is more effective than single evaluations of diastolic or systolic function for assessing macaque cardiac function.^8^, ^9^ The results showed that the Tei index increased during the e‐RA stage, and there was a significant difference in the a‐RA stage compared with normal macaques at the same stage (Figure 6C). This suggests that abnormal changes in cardiac diastolic and systolic function occur as early as the e‐RA stage. The E/A ratio (ratio of early diastolic peak velocity [E] to late diastolic peak velocity [A]) of blood flow at the mitral and tricuspid valve orifices was simultaneously detected. An E/A < 1 indicates abnormal cardiac diastolic function in macaques. Among these parameters, the mitral valve E/A ratio reflects left ventricular diastolic function, and the tricuspid valve E/A ratio reflects right ventricular diastolic function.^10^, ^11^ Five CIA macaques (XX, ZZ, TT, YY, LL) had diastolic dysfunction: 1 (LL) in the IIR stage, 3 (XX, TT, YY) in the e‐RA stage, and 1 (ZZ) in the a‐RA stage. Tricuspid valve damage was more severe, suggesting that RA affects right ventricular diastolic function (Figure 6D,E, Table S5). However, no significant changes in cardiac systolic function were observed (Figure 6F,G). In the model group, systolic blood pressure increased significantly during the a‐RA stage, showing a significant difference compared to the normal group (Figure 6H). However, diastolic blood pressure did not change significantly (Figure 6I). Based on the above results, valve damage in CIA macaques primarily occurs in the tricuspid valve, and right ventricular diastolic function is more significantly affected.

**FIGURE 7:**
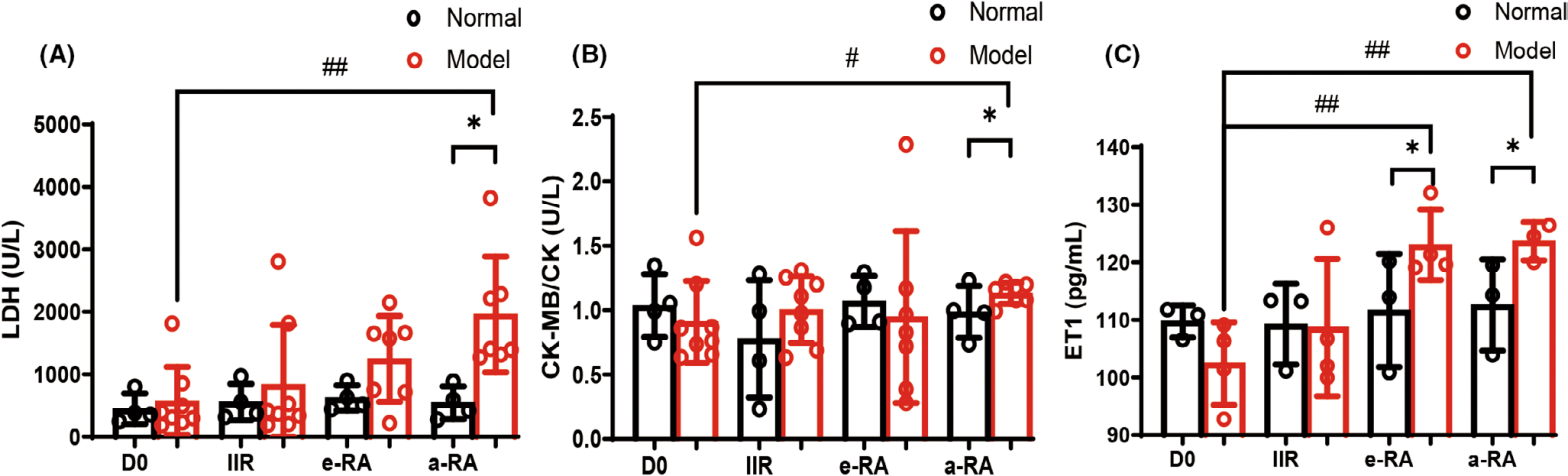
Cardiac systolic function of macaques and related indicators of peripheral blood cardiac and vascular injury. (A) Lactate dehydrogenase. (B) Creatinekinase‐MB/Creatine kinase. (C) Endothelin‐1. ^#^ *p* < 0.05, ^##^ *p* < 0.01 as compared with the D0 Model group. *p < 0.05, as compared with the e‐RA/a‐RA Normal group.

To determine when myocardial damage initiates, we measured the levels of myocardial damage markers (lactate dehydrogenase [LDH], creatine kinase [CK], and creatine kinase‐MB [CK‐MB]) in macaque peripheral blood at different stages.^12^, ^13^, ^14^ The results indicated that LDH levels and the CK‐MB/CK ratio began to increase significantly at the a‐RA stage, with significant differences compared to the normal group (Figure 7A,B). This provides further evidence that RA induces myocardial damage. To identify the onset of vascular injury, we measured endothelin‐1 (ET‐1) levels in macaque peripheral blood.^15^ The results showed that peripheral blood ET‐1 levels increased significantly during the e‐RA stage (Figure 7C), suggesting that vascular damage may occur before myocardial damage. We also found that RA is highly likely to induce pulmonary inflammation (Figure S1, Table S6). Whether this phenomenon intensifies with disease progression requires further verification. However, based on respiratory rate monitoring results, the respiratory rate of CIA macaques increased significantly with disease progression (Figure S2), confirming that RA impacts lung function.

## 4 DISCUSSION

### 4.1 Evaluation and staging of the CIA macaque

It is well established that common NHPs (e.g., macaques, cynomolgus monkeys, marmosets) play an critical role in research fields such as infectious immune responses, immune aging, and autoimmune diseases, serving as a bridge between basic research and clinical applications.^16^, ^17^ The CIA model is considered the most similar to human RA pathogenesis. This study is the first to establish a CIA macaque model and to continuously monitor disease progression in the same individuals. Based on the 2010 EULAR/ACR early RA diagnostic criteria and the 2016 EULAR RA staging recommendations, we employed MRI, CT, and ultrasound to evaluate CIA macaques across four dimensions: joint swelling, serology, acute‐phase reactants, and global scores. We observed that ESR and CRP levels began to rise significantly by D14 after the first immunization. Despite individual variability, the overall trend was clear. After the second immunization on D21, two RA‐specific markers (RF and ACPA) started to increase from D28 and D35, respectively. Foot swelling also appeared on D35, with six of eight CIA macaques scoring ≥6 on the global assessment. Thus, we designated D35 as the onset of the early RA (e‐RA) stage. Given that ACPA levels rose closer to the e‐RA stage and considering individual differences, we analyzed RF and ACPA changes in each macaque. One macaque (GZ) that did not reach a score of 6 showed minimal ACPA elevation but similar RF trends to other macaques, supporting the higher specificity of ACPA over RF in RA diagnosis (Figure S3)—a finding consistent with clinical observations.^18^, ^19^, ^20^ Next, we defined the IIR stage. ESR and CRP levels began to rise significantly by D14, peaking on D28 and D35, respectively. IgA and IgM levels increased from D14, while IgG and IgD levels began to rise on D21 and D28, respectively, peaking on D49. These findings suggest that IgA and IgM began to rise before the second immunization, IgG started to increase on the day of the second immunization, and IgD began to rise 7 days after the second immunization. These inflammatory and immune responses persisted beyond D35. Although no overt joint swelling was observed during this period, MRI and ultrasound detected minor joint edema, synovial hyperplasia, and blood flow signals, leading us to classify D14–D34 as the IIR stage.

The study's focus was on determining the onset and duration of the a‐RA stage. Per ACR criteria, the key distinction between a‐RA and e‐RA is the presence of bone erosion. CT and X‐ray revealed bone erosion and joint space narrowing starting on D42, marking the onset of a‐RA. ESR and CRP levels peaked on D28 and D35, respectively, then declined, normalizing by D70. RF and ACPA peaked on D63 and D56, respectively, before decreasing—though levels remained elevated compared to baseline on D70. Joint swelling in CIA macaques lasted from D35 to D63, resolving by D70. Thus, we defined D42–D70 as the a‐RA stage and D35–D41 as the e‐RA stage. Establishing this evaluation and staging system for the CIA macaque model provides a robust framework for studying humanized monoclonal antibodies and deepens our understanding of RA progression.

### 4.2 Bone erosion in CIA macaques exhibits partial irreversibility

Bone erosion is a critical challenge in RA treatment. Clinically, the focus is on alleviating active RA symptoms (e.g., joint swelling and elevated inflammatory markers). CT revealed bone erosion starting on D42, with increasing severity over time. By D70, acute‐phase reactants and serological markers had normalized, and swelling had subsided. However, comparing D56 and D100, bone erosion showed no improvement and even worsened in some cases. X‐rays also revealed progressive joint space narrowing and surface roughness. Peripheral blood analysis of bone destruction markers showed stable OPG levels, while RANKL levels rose during e‐RA, peaked in a‐RA, and remained elevated on D100 compared to baseline. These findings suggest that bone erosion persists despite the resolution of a‐RA symptoms, indicating partial irreversibility.

### 4.3 IgD levels correlate closely with RA progression

Research on immunoglobulins in RA has focused on IgA, IgM, and IgG, with studies confirming their elevated levels in RA patients and their role in symptom alleviation when reduced.^21^, ^22^, ^23^ These immunoglobulins are often studied alongside RF, with the proportions of IgA RF, IgG RF, and IgM RF in RA patients reported as 43%, 33%, and 57%, respectively,^24^ indicating their lack of specificity. IgD has been less studied in RA. Our team previously found elevated peripheral blood IgD levels in RA patients, which promoted mononuclear cell activation and accelerated disease progression. The expression of the IgD Fc receptor on RA fibroblast‐like synoviocytes (FLS) further suggests IgD as a potential therapeutic target.^25^ Subsequent animal studies demonstrated that IgD‐Fc‐Ig effectively treated rodent models of RA, systemic lupus erythematosus (SLE), and leukemia by inhibiting IgD‐IgDR‐Lck signaling and suppressing T and B cell activation.^26^, ^27^, ^28^ This study revealed that IgA, IgM, and IgG levels rose before the second immunization, while IgD increased only after D28 (7 days post‐second immunization on D21). Unlike other immunoglobulins, IgD trends closely mirrored RA progression (Figure S4). Individual analysis of CIA macaques showed that GZ (global score <6) had transient IgD elevation, peaking on D42 and normalizing by D70. Additionally, macaques in group A (score >7) had significantly higher peak IgD levels on D49 compared to group B (score <7), confirming that IgD levels correlate with disease severity (Figure S5). Thus, this study extends prior findings by delineating peripheral blood IgD dynamics in an NHP RA model, supporting the use of IgD fusion proteins as a preclinical therapeutic strategy.

The pathogenesis of RA is complex. Though it presents as joint lesions, RA is essentially a systemic inflammatory immune response involving numerous immune cells and inflammatory factors. The heart is the most frequently involved organ: the cardiovascular morbidity rate of RA patients is 1.5–2 times higher than that of the general population, and cardiovascular mortality accounts for approximately 10%–30% of total RA‐related mortality.^29^, ^30^ The pathogenesis of RA is complex. Though it presents as joint lesions, RA is essentially a systemic inflammatory immune response involving numerous immune cells and inflammatory factors. Changes in cardiac function during RA pathogenesis are rarely reported but critical for early intervention. CIA macaques showed slight elevation of systolic/diastolic blood pressure in a‐RA; the ultrasound‐derived Tei index (calculated from isovolumic contraction time [IVCT], isovolumic relaxation time [IVRT], and atrial ejection time [AET]) increased during the e‐RA stage and differed significantly from controls in a‐RA. No changes in systolic function were detected via ejection fraction (EF) or fractional shortening (FS), indicating that abnormal cardiac diastolic function begins in the e‐RA stage. We observed the E/A ratio of the mitral and tricuspid valves and found that 5 CIA macaques had abnormal diastolic function, with more severe tricuspid valve damage—suggesting that RA preferentially affects right ventricular diastolic function. We also observed valvular regurgitation and echo enhancement in the macaques: 8 and 4 CIA macaques had valvular regurgitation and echo enhancement, respectively, with nearly half of these cases occurring in the e‐RA stage and primarily involving the tricuspid valve. To determine when myocardial damage initiates, we measured the levels of myocardial damage markers (LDH, CK, and CK‐MB) in CIA macaques at different stages. Notably, the CK‐MB/CK ratio is a specific parameter for evaluating the relative contribution of cardiomyocyte damage (compared to non‐specific myocardial injury).^31^, ^32^, ^33^, ^34^ The results showed that LDH levels and the CK‐MB/CK ratio began to increase in the a‐RA stage, and systolic/diastolic blood pressure increased in the a‐RA stage—collectively confirming RA‐induced cardiac damage. Endothelin‐1 (ET‐1) is a potent vasoconstrictor that regulates vascular contraction together with nitric oxide (NO). Abnormal vascular function is associated with increased ET‐1 expression. We found that peripheral blood ET‐1 levels in CIA macaques increased significantly during the e‐RA stage, suggesting that vascular damage begins in the e‐RA stage. Our findings demonstrate that cardiac valvular damage in CIA macaques primarily affects the tricuspid valve, with more evident impairment of right ventricular diastolic function. Furthermore, peripheral vascular injury appears to precede cardiac involvement—consistent with previous reports.^35^, ^36^

## 5 CONCLUSION

This study was based on the 2010 EULAR/ACR joint early RA diagnostic criteria. It established the first evaluation criteria for NHP RA models. By detecting acute‐phase reactants and serological indicators, and using techniques such as MRI, CT, and ultrasound to monitor the entire disease course in NHPs, we divided the progression of CIA macaques into three stages: the IIR stage (D14–D34), the e‐RA stage (D35–D41), and the a‐RA stage (D42–D70). This is also the first international study to simulate RA pathogenesis and continuously monitor the disease course in individual NHPs. This research avoids limitations such as the impracticality of timely detection of indicators during human RA onset and physiological/pathogenic differences between humans and rodents. This study provides experimental evidence for identifying early indicator changes during RA development and further explores the pathological mechanisms of RA, as well as the potential of humanized biological molecules as primary RA therapeutics.

## AUTHOR CONTRIBUTIONS

**Lei Zhang:** Data curation; formal analysis; writing – original draft. **Haifeng Jiang:** Investigation; methodology; supervision. **Zhen Xu:** Investigation; methodology; resources. **Tingyu Dong:** Investigation; validation; visualization. **Xiaoyi Liu:** Investigation; validation; visualization. **Shangxue Yan:** Investigation; validation. **Jiajie Kuai:** Investigation; validation. **Yan Chang:** Investigation; validation. **Wei Wei:** Conceptualization; funding acquisition; supervision; writing – review and editing.

## FUNDING INFORMATION

This work was supported by the National Natural Science Foundation of China (Nos. 81973332, 82173824).

## CONFLICT OF INTEREST STATEMENT

The authors declare that they have no competing interests.

## ETHICS STATEMENT

The research adhered to the principles of China's Laboratory Animal Care Guidelines and was approved by the Ethics Review Committee for Animal Experimentation of the Institute of Clinical Pharmacology, Anhui Medical University (approval number: PT‐2020‐001).

## Supporting information


Data S1.

## Data Availability

The data that support the findings of this study are available from the corresponding author upon reasonable request.
