# Supplementary material for: The characteristics of collagen‐induced rheumatoid arthritis in macaques and the changes of heart
Source: Animal Model Exp Med. 2026 Mar 17;9(3):609–20. doi: 10.1002/ame2.70185 (PMC13176097; doi:10.1002/ame2.70185)
Supplement: Supplementary file 1 — Data S1. [file AME2-9-609-s001.docx]

**Supplementary materials**

**The characteristics of collagen-induced rheumatoid arthritis in macaques**

**and the changes of heart**

Lei Zhang^1,2^, Haifeng Jiang^1^, Zhen Xu^1^, Tingyu Dong^1^, Xiaoyi Liu^1^, Shangxue Yan^1^, Jiajie Kuai^1^, Yang Chang^1^, and Wei Wei^1^

^1^School of Pharmacy and Science, Anhui Medical University; Key Laboratory of Anti-inflammatory and Immune Medicine, Ministry of Education; Institute of Clinical Pharmacology, Anhui Medical University, Hefei, Anhui, 230032, China;

^2^Department of pharmacy, The First Affiliated Hospital of Anhui Medical University, Anhui Public Health Clinical Center, Hefei, Anhui, 230012, China

^*^ **Correspondence should be addressed to:**

Wei Wei

Prof. Wei Wei, School of Pharmacy and Science, Institute of Clinical Pharmacology, Anhui Medical University, Hefei, Anhui, 230032, China, Office Phone/Fax: +86-551-6516-1209, wwei@ahmu.edu.cn

**1 Materials and Methods**

**1.1 The naming rules of macaques**

Based on the initials of the research team members and the descriptions of macaques in ancient Chinese poetry, we named the experimental macaques to show respect for the experimental subjects. Normal group (FF, QS, CF, MY), Model group (TT, ZZ, YY, XX, LL, TF, GZ, RF).

**1.2 Enzyme-linked immunosorbent assay (ELISA)**

Macaques peripheral blood was collected in a pro-coagulation tube and centrifuged at 1500 rpm for 15 min, and the collected supernatant served as the serum required for the experiment. Measure with ELISA kit (Shanghai Enzyme-linked Biotechnology Co., Ltd.）according to standard procedure. Finally, the absorbance of the culture supernatants was recorded at a wavelength of 450 nm using a microplate reader. Testing indicators include immunoglobulin D (IgD），anti-citrullinated protein antibodies (ACPA)，Receptor activator of nuclear factor kappa-B ligand (RANKL)，osteoprotegerin (OPG)，Endothelin-1(ET-1)，Creatine kinase (CK)，Creatine kinase isoenzyme (CK-MB).

**1.3 Blood parameters**

Complete blood count (Automatic blood analyzer XE-2100, Sysmex Corporation，GREG) and serum chemistry analysis were conducted (Automatic biochemical analyzer Cobas8000, Roche Diagnostics CH; Automatic biochemical analyzer BS-830,CHN); erythrocyte sedimentation rate（ESR），C-reactive protein（CRP）, Rheumatoid factor (RF), immunoglobulin G (IgG), immunoglobulin M（IgM）, immunoglobulin A (IgA) were measured.

**2. Supplementary Results**

**2.1 Diagnostic Criteria for RA in Laboratory Animals**

**TableS1** **Diagnostic Criteria for RA in Laboratory Animals^a^**

|  | Indicators | Score |
| --- | --- | --- |
| Joint involvement^b^ | Medium and large joints^c^ 1 | 0 |
|  | Medium and large joints 2-4 | 1 |
|  | Small Joint^d^ 1-3 | 2 |
|  | Small Joint 4-10 | 3 |
|  | > 10 (At least one small joint) | 5 |
| Serology^e^ | Autoantibody negative | 0 |
|  | Low titer autoantibody positivity (with antibody levels exceeding the normal range by 10% - 30%) | 2 |
|  | High titer autoantibody positivity (antibody levels exceeding the normal range by more than 30%) | 3 |
| Acute reactants | Normal CRP and ESR levels | 0 |
|  | Elevated CRP or ESR levels | 1 |
| Overall Body Score | 1-3 | 0 |
|  | 4-8 | 1 |

Note: ^a^ Diagnosis of RA model experimental animals according to the following criteria score ≧ 6/10.^b^ Joint involvement refers to any joint that is red and swollen, which can be confirmed by medical imaging evidence. ^c^ Medium and large joints refer to wrist，ankle and knee joints.^d^ Small joints refer to finger (toe) joints. ^e^Serology refers to ACPA or RF

**2.2 Scoring Criteria for Laboratory Animal RA**

**Table S2 Scoring Criteria for Laboratory Animal RA**

| No. | Criterion | Score |
| --- | --- | --- |
| 1 | Elevated ESR or CRP | 1 |
| 2 | Meet criterion 1, and have an increase of 10% to 30% in RF or ACPA. | 3 |
| 3 | Meet criterion 2, and have swelling in 2 to 4 medium or large joints;  Meet criterion 1, and have an increase of RF or ACPA by more than 30%. | 4 |
| 4 | Meet criterion 2, and have swelling in 1 to 3 small joints. | 5 |
| 5 | Meet criterion 2, and have swelling in 2 to 4 medium or large joints, and 1 to 3 small joints.  Meet criterion 2, and have swelling in 4 to 10 small joints. | 6 |
| 6 | Meet criterion 2, and have swelling in 4 to 10 small joints, with at least 2 swollen small joints in each of the metacarpophalangeal and metatarsophalangeal joints;  Meet criterion 3, and have swelling in 4 to 10 small joints. | 7 |
| 7 | Meet criterion 3, and have swelling in 4 to 10 small joints, with at least 2 swollen small joints in each of the metacarpophalangeal and metatarsophalangeal joints. | 8 |
| 8 | Meet criterion 2 and have more than 10 joints (including at least 1 small joint). | 9 |
| 9 | Meet criterion 1, and RF or ACPA increase by more than 30%, and involve more than 10 joints (including at least 1 small joint). | 10 |

**2.3 Statistical table of valvular blood regurgitation in different stages of CIA macaques**

**Table S3 Statistical table of valvular blood regurgitation in different stages of CIA macaques (n=7)**

| Blood reflux | D0 | | | IIR | | | e-RA | | | a-RA | | |
| --- | --- | --- | --- | --- | --- | --- | --- | --- | --- | --- | --- | --- |
|  | MV | TV | AV | MV | TV | AV | MV | TV | AV | MV | TV | AV |
| TT | - | - | **-** | - | - | **-** | - | - | **-** | + | + | + |
| ZZ | - | - | - | - | - | - | - | - | - | + | - | + |
| XX | - | - | - | - | - | - | - | + | - | - | + | - |
| YY | - | - | - | - | - | - | - | + | - | / | / | / |
| LL | - | - | - | - | - | - | + | - | - | + | + | - |
| TF | - | - | - | - | - | - | - | - | - | - | + | - |
| GZ | - | - | - | - | - | - | - | - | - | - | + | - |

Note: “-”represents no regurgitation, “+”represents regurgitation，“/”represents no data detected. MV tricuspid valv, TV tricuspid valv, AV aortic valve.

**2.4 Statistical table of echo enhancement of CIA macaques valve at different stages**

**Table S4 Statistical table of echo enhancement of CIA macaques valve at different stages(n=7)**

| echo enhancement | D0 | | | IIR | | | e-RA | | | a-RA | | |
| --- | --- | --- | --- | --- | --- | --- | --- | --- | --- | --- | --- | --- |
|  | MV | TV | AV | MV | TV | AV | MV | TV | AV | MV | TV | AV |
| TT | + | - | **-** | + | - | **-** | + | - | + | + | + | + |
| ZZ | + | - | - | + | - | - | + | - | + | + | - | + |
| XX | + | - | - | + | - | + | + | - | + | + | + | + |
| YY | + | - | - | + | - | + | + | - | + | / | / | / |
| LL | + | - | - | + | - | - | + | - | - | + | - | - |
| TF | + | - | - | + | - | - | + | - | - | + | - | - |
| GZ | + | - | - | + | - | - | + | - | - | + | - | - |

Note: “-”represents no echo enhancement, “+”represents echo enhancement，“/”represents no data detected.MV tricuspid valv, TV tricuspid valv, AV aortic valve.

**2.5 E/A ratios of mitral and tricuspid valves in CIA macaques at different stages**

**Table S5 E/A ratios of mitral and tricuspid valves in CIA macaques at different stages(n=7)**

| E/A | D0 | | IIR | | e-RA | | a-RA | |
| --- | --- | --- | --- | --- | --- | --- | --- | --- |
|  | MV | TV | MV | TV | MV | TV | MV | TV |
| XX | ＞1 | ＞1 | ＞1 | ＞1 | ＞1 | ＜1 | ＞1 | ＞1 |
| ZZ | ＞1 | ＞1 | ＞1 | ＞1 | ＞1 | ＞1 | ＜1 | ＜1 |
| TT | ＞1 | ＞1 | ＞1 | ＞1 | ＜1 | ＜1 | ＞1 | ＞1 |
| YY | / | ＞1 | / | / | / | ＜1 | / | / |
| LL | ＞1 | ＞1 | ＞1 | ＜1 | / | / | ＞1 | ＞1 |
| TF | ＞1 | ＞1 | ＞1 | ＞1 | ＞1 | ＞1 | ＞1 | ＞1 |
| GZ | ＞1 | ＞1 | ＞1 | ＞1 | ＞1 | ＞1 | ＞1 | ＞1 |

Note: E/A≥1 is normal, and E/A<1 is abnormal ,“/”represents no data detected.MV tricuspid valv, TV tricuspid valv.

**2.6 Statistical chart of CT changes of CIA macaques at different stages**

**Table S6 Statistical chart of CT changes of CIA macaques at different stages(n=8)**

| Name | D0 | IIR | e-RA | a-RA |
| --- | --- | --- | --- | --- |
| ZZ | **/** | **+** | **++** | **+++** |
| TT | **/** | **/** | **+** | **+** |
| XX | **/** | **/** | **+** | **+** |
| YY | **/** | **+** | **+** | **_** |
| RF | **/** | **+** | **/** | **/** |
| GZ | **/** | **/** | **+** | **/** |
| TF | **/** | **+** | **+** | **+** |
| RF | **/** | **/** |  |  |

Note:“/”represents no small nodules or diffuse shadows, “+”represents the degree of small nodules or diffuse shadows, “-” represents sacrifice

**2.8 CT observation of pulmonary inflammatory changes in CIA macaques at different stages**

D0 represents the normal period of the macaque. IIR shows diffuse small nodular shadows in the lungs. In e-RA, the nodular shadows in both lungs increase in size and number, and some present as patchy dense shadows. In a-RA, reticular shadows and tractional bronchiectasis or bronchiolitis are observed (Figure S1).


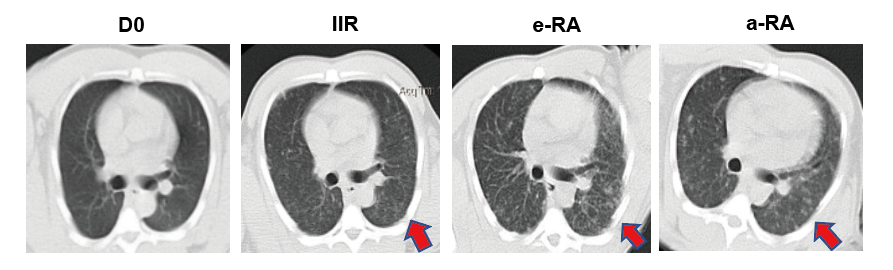


**Figure S1 CT observation of pulmonary inflammatory changes in CIA macaques at different stages.**

**2.9 The changes in the respiratory rate of macaques at different periods**

FigureS2 shows as the disease progresses, the respiratory rate of the model group macaques shows an increasing trend, which is significantly higher compared to the normal group. Figure S2B indicates that from the changes in the respiratory rate of individual macaques, it can be seen that the model group (colored segments) has an upward trend, while the normal group (black segments) shows almost no change.

**
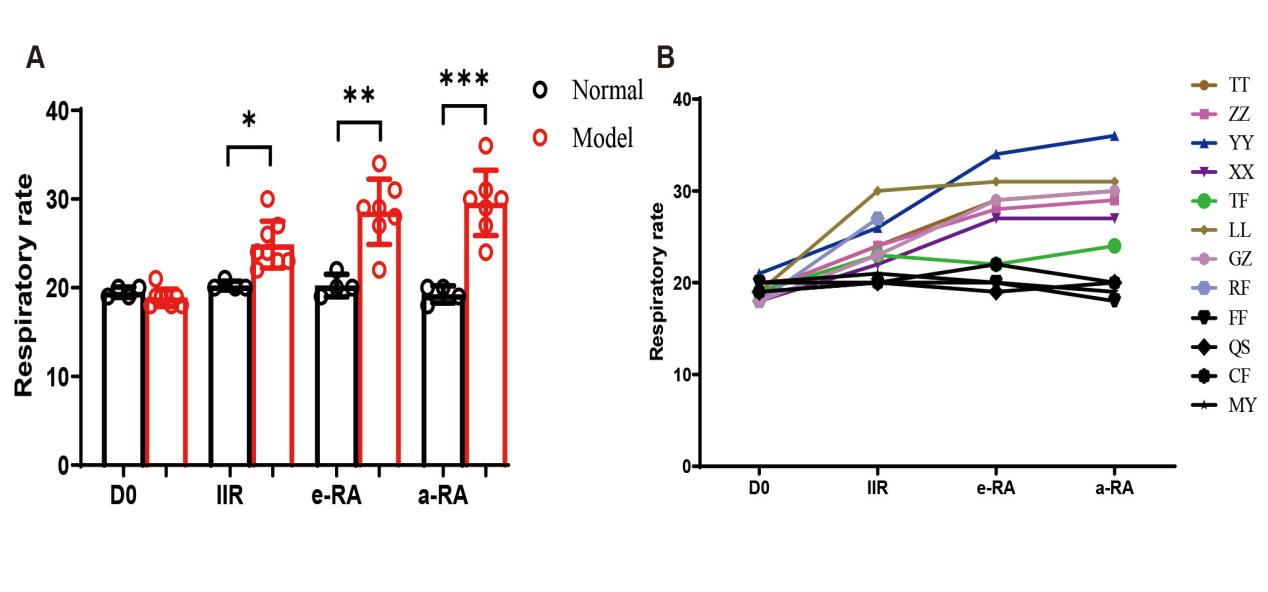
**

**Figure S2** **The changes in the respiratory rate of macaques at different periods.** (A)Bar chart of changes in respiratory rate; (B)Line graph of changes in respiratory rate. ^*^*P*<0.05, ^**^*P*<0.01 ^***^*P*<0.001 compared with the Normal group.

**2.10 Changes in peripheral blood RA-related diagnostic indicators in CIA monkeys at different times**

Figure S3 analyzes which group is more specific by observing the changing trends of RF and ACPA levels in each model group of macaques.


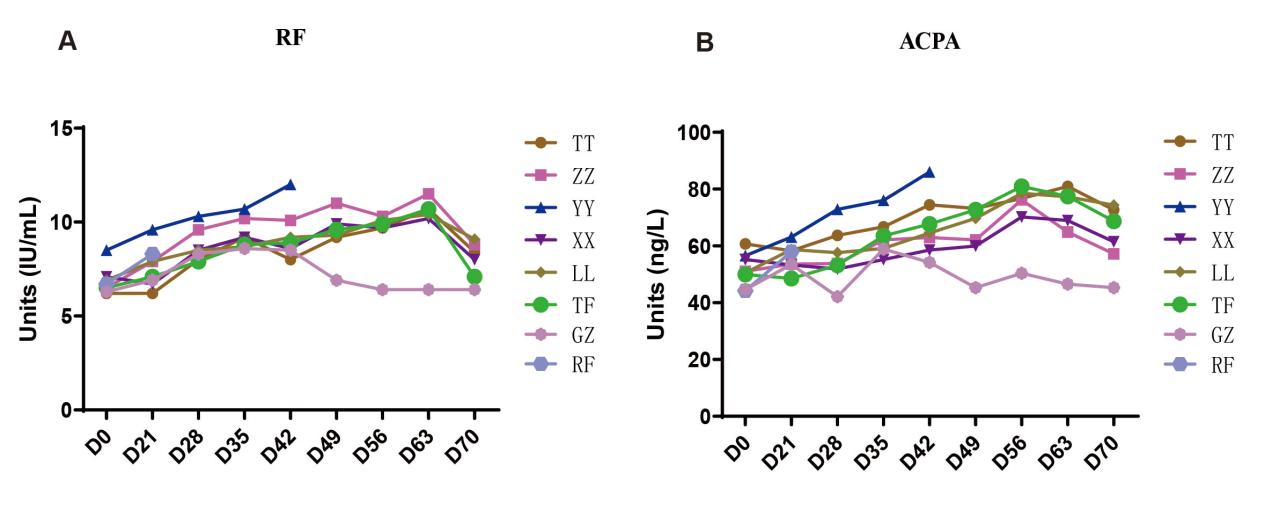


**Figure S3 Line graph showing the changes in RA-related diagnostic indicators in the peripheral blood of CIA monkeys at different times.** (A) Rheumatoid factor; (B) Anti-citrullinated protein antibodies.

**2.11 Correlation analysis of the levels of immunoglobulins and the scores**

Through the correlation analysis of immunoglobulin levels and disease severity scores, it was found that the levels of IgD, IgA, IgG, and IgM were all positively correlated with the severity of the disease. Among them, the level of IgD had the strongest correlation with the severity of the disease (Figure S4).


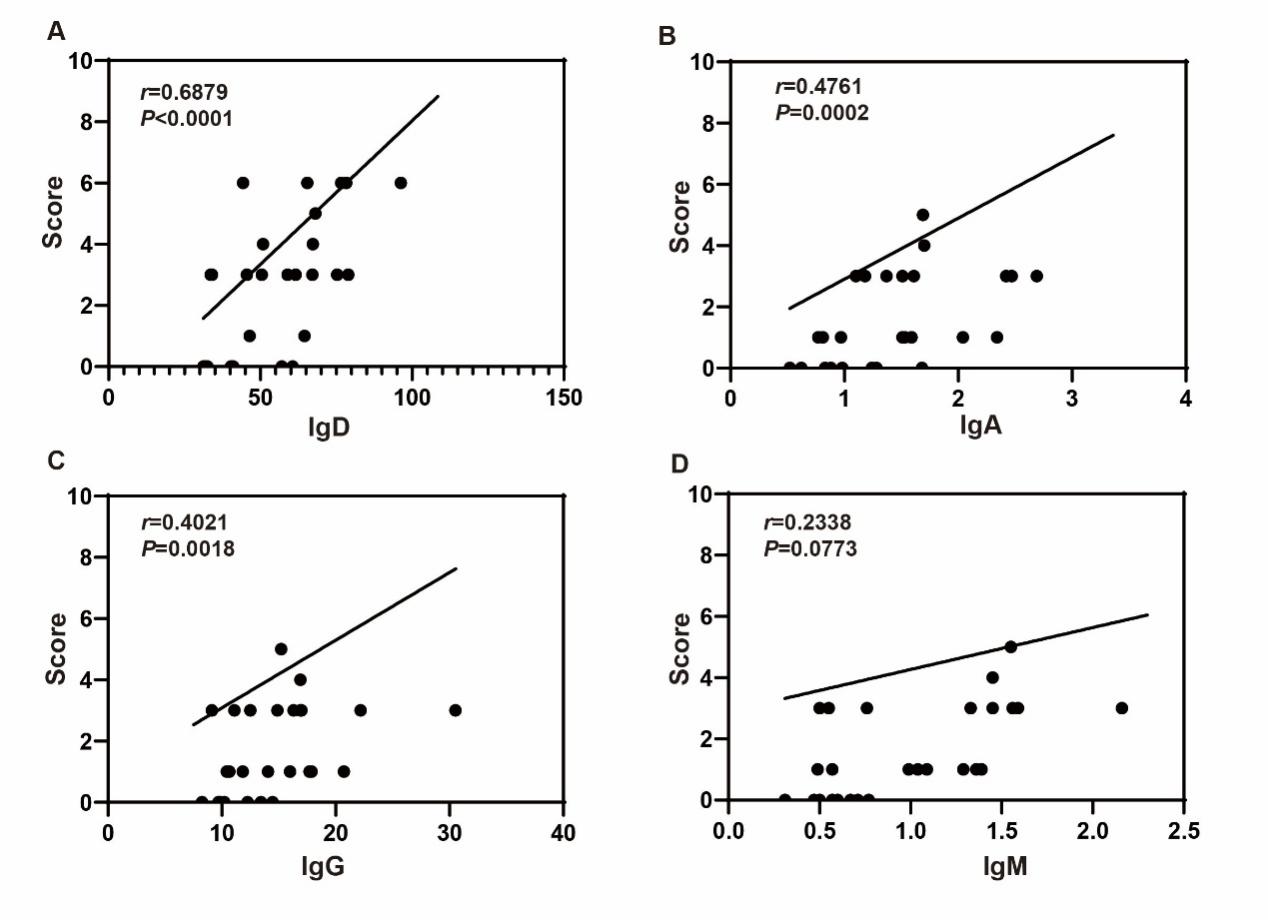
**Figure S4 Correlation analysis of the levels of immunoglobulins and the scores**

**2.12** **Changes in serum immunoglobulin levels in peripheral blood of different macaque in the CIA group**

Figures S5A, B, C, D are used to observe the differences in immunoglobulin levels between macaques with a score less than 6 (GZ) and other macaques with a score greater than 6. Figure S5E is used to observe whether the level of IgD is related to the severity of the disease.


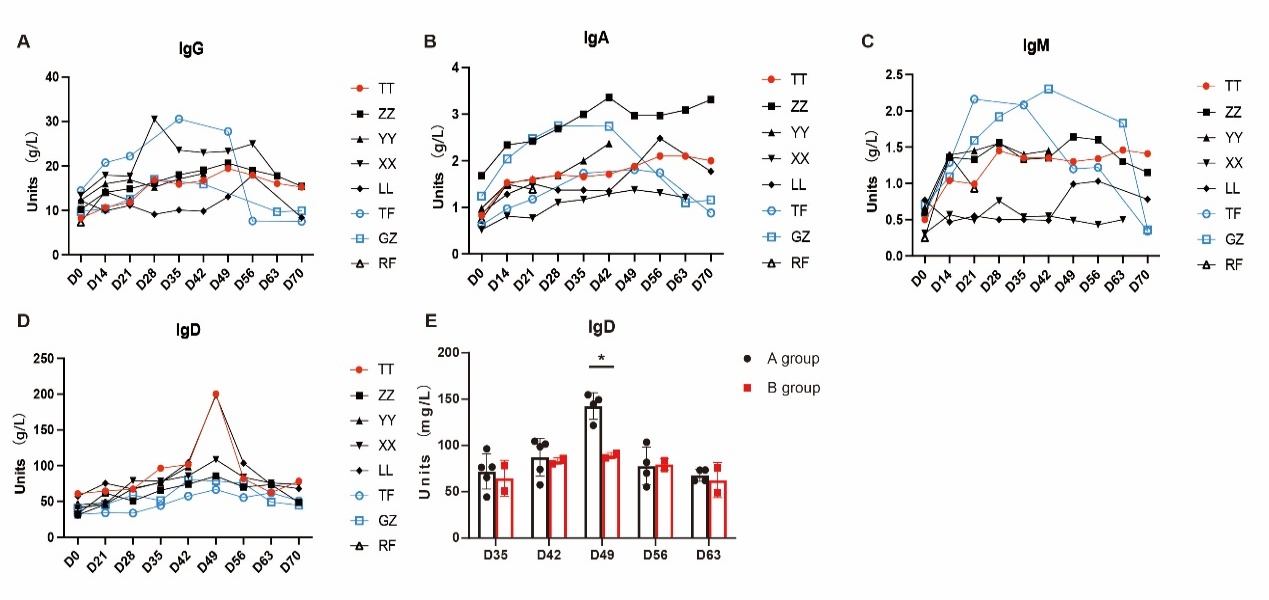


**Figure S5 Changes in serum immunoglobulin levels in peripheral blood of different macaque in the CIA group.** (A) Changes of IgG levels in different macaque monkeys in the CIA group(D0-D70); (B) Changes of IgA levels in different macaque monkeys in the CIA group(D0-D70); (C) Changes of IgM levels in different macaque monkeys in the CIA group(D0-D70); (D) Changes of IgD levels in different macaque monkeys in the CIA group(D0-D70); (E) Changes of IgD levels in group A and group B in D35-D63. ^*^*P*<0.05 compared with the A group.
